# BpfD Is a c-di-GMP Effector Protein Playing a Key Role for Pellicle Biosynthesis in *Shewanella oneidensis*

**DOI:** 10.3390/ijms25179697

**Published:** 2024-09-07

**Authors:** Jean-Pierre Poli, Anne Boyeldieu, Alexandre Lutz, Amélie Vigneron-Bouquet, Amine Ali Chaouche, Marie-Thérèse Giudici-Orticoni, Michel Fons, Cécile Jourlin-Castelli

**Affiliations:** 1 Aix Marseille Univ, CNRS, BIP, Marseille, France; poli_jp@univ-corse.fr (J.-P.P.); anne.boyeldieu@univ-tlse3.fr (A.B.); alexandre-lutz@etud.univ-tln.fr (A.L.); avigneron@imm.cnrs.fr (A.V.-B.); aalichaouche@imm.cnrs.fr (A.A.C.); giudici@imm.cnrs.fr (M.-T.G.-O.); mfons@imm.cnrs.fr (M.F.); 2 UMR CNRS 6134 Laboratoire Sciences pour l’Environnement (SPE), Université de Corse, Corte, France; 3 Université de Toulon, MAPIEM, Toulon, France

**Keywords:** biofilm, pellicle, diguanylate cyclase, secondary messenger, c-di-GMP, regulatory network, *Shewanella*

## Abstract

The aquatic γ-proteobacterium *Shewanella oneidensis* is able to form two types of biofilms: a floating biofilm at the air–liquid interface (pellicle) and a solid surface-associated biofilm (SSA-biofilm). *S. oneidensis* possesses the Bpf system, which is orthologous to the Lap system first described in *Pseudomonas fluorescens*. In the Lap systems, the retention of a large adhesin (LapA) at the cell surface is controlled by LapD, a c-di-GMP effector protein, and LapG, a periplasmic protease targeting LapA. Here, we showed that the Bpf system is mandatory for pellicle biogenesis, but not for SSA-biofilm formation, indicating that the role of Bpf is somewhat different from that of Lap. The BpfD protein was then proved to bind c-di-GMP via its degenerated EAL domain, thus acting as a c-di-GMP effector protein like its counterpart LapD. In accordance with its key role in pellicle formation, BpfD was found to interact with two diguanylate cyclases, PdgA and PdgB, previously identified as involved in pellicle formation. Finally, BpfD was shown to interact with CheY3, the response regulator controlling both chemotaxis and biofilm formation. Altogether, these results indicate that biofilm formation in *S. oneidensis* is under the control of a large c-di-GMP network.

## 1. Introduction

Bacteria can adopt two lifestyles. They live either as planktonic cells able to move independently in their environment or as sessile cells forming a community called a biofilm. In nature, bacteria are mainly found in biofilms [1]. Several forms of biofilms have been described. Bacteria can adhere to biotic or abiotic surfaces forming what is called a solid surface-associated biofilm (SSA-biofilm) [2,3]. They can also form a floating biofilm at the air–liquid interface, also named a pellicle [4,5]. A biofilm is a community of cells encased in a self-produced extracellular matrix. The composition of the matrix can vary from one bacterium to another and can also differ from one type of biofilm to another even for the same bacterium. Nevertheless, the main components of the matrix are exopolysaccharides, extracellular proteins, and DNA [6,7].

The transition from planktonic to sessile lifestyles is governed by several molecular actors. A key player is the cyclic di-guanosine monophosphate (c-di-GMP), a secondary messenger involved not only in the regulation of biofilm formation but also in the regulation of motility, virulence, and differentiation [8,9,10]. The concentration of c-di-GMP in the cells is fine-tuned by a whole array of enzymes. C-di-GMP is synthetized from GTP by diguanylate cyclases (DGCs) and hydrolyzed by phosphodiesterases (PDEs). The DGCs are characterized by GGDEF domains containing the consensual motif GG(D/E)EF crucial for catalytic activity. They function as dimers in which each subunit binds a GTP molecule. Most DGCs also contain an inhibitory site (RXXD) located five residues upstream of the catalytic site. The fixation of c-di-GMP to this inhibitory site allows negative feedback control of DGC activity. There are two types of PDEs: the ones containing an EAL domain and hydrolyzing c-di-GMP into pGpG, and the others harboring an HD-GYP domain and hydrolyzing c-di-GMP into GMP. Once c-di-GMP is synthetized, it can interact with a third type of actor, the effectors, to induce a cellular response. Effectors can be RNA or proteins [11]. The effector proteins are less characterized than DGCs or PDEs and can contain different types of domains (PilZ, YadQ, etc.). In some cases, effector proteins contain degenerated GGDEF or EAL domains, in which the catalytic sites are not conserved, giving rise to proteins unable to synthetize or hydrolyze c-di-GMP but still able to bind to it.

A well-characterized system involved in biofilm formation and controlled by c-di-GMP is the Lap system of Pseudomonas fluorescens [12]. The main components of this system are LapA, an adhesin secreted to the cell surface by a type-I secretion system (LapBCE) and involved in cell-surface adhesion; LapG, a periplasmic protease targeting LapA; and LapD, an effector protein containing degenerated GGDEF and EAL domains and binding to c-di-GMP. In biofilm conditions, LapD receives c-di-GMP from a specific DGC GcbC [13]. LapD bound to c-di-GMP becomes active and can sequester the LapG protease. This latter is then unable to cleave LapA, leading to LapA accumulation at the surface of the cells and promoting cell-surface interaction. This constitutes the first step of biofilm formation. The Lap systems have been described in several bacteria belonging to different species such as Pseudomonas, Legionella, Bordetella, Vibrio, and Shewanella [14,15,16,17,18].

*Shewanella oneidensis* is a motile aquatic γ-proteobacterium. It was shown to form two types of biofilms: an SSA-biofilm when cultivated in agitated/aerated minimal medium and a pellicle when cultivated in static rich medium [19,20,21,22]. A cluster of genes (*so_4317* to *so_4323*) was previously shown to encode proteins sharing similarities to the Lap proteins of *P. fluorescens* [15]. The system of *S. oneidensis* was named Bpf (Biofilm-Promoting Factor) and contains BpfA (SO_4317), a homolog of the adhesin LapA; BpfD (SO_4323), a homolog of the effector LapD; BpfG (SO_4322), the unique homolog of LapG in *S. oneidensis*; and a type-I secretion system composed of AggC (SO_4318, homologous to LapB), AggB (SO_4319, homologous to LapC), and AggA (SO_4320, homologous to LapE). A mutant of *bpfA* and a mutant of either *aggA*, *aggB,* or *aggC* were reported to be totally impaired in biofilm formation, while a mutant of *bpfG* was greatly affected but not totally impaired and a *bpfD* mutant was only partially impaired [15,23]. It is noteworthy that the conditions used to perform these tests were not discriminant enough to distinguish the SSA-biofilm from the pellicle. It was hypothesized that the Bpf system of *S. oneidensis* is an ortholog of the Lap system of *P. fluorescens* since the BpfA adhesin was proved to interact with the BpfG protease, which itself interacts with the BpfD protein [15]. Even though c-di-GMP was shown to be required for biofilm formation in *S. oneidensis* like in other bacteria [22,24], it is not currently known whether BpfD is able to bind c-di-GMP or not and, if it is the case, whether BpfD receives c-di-GMP from a specific DGC or not. Several DGCs were identified to play a role in biofilm formation in *S. oneidensis*. Two DGCs, PdgA and PdgB, were found to restore pellicle formation in the *cheY3* pellicle-deficient mutant [24]. A similar approach, used in the context of SSA-biofilm formation, led to the identification of two additional DGCs [25]. Interestingly, three out of these four DGCs interact with CheY3, the response regulator controlling both chemotaxis and biofilm formation in *S. oneidensis*. Moreover, the complex regulatory network governing biofilm formation and centered around CheY3 also contains an effector protein, MxdA. MxdA was shown to bind c-di-GMP and to interact with both CheY3 and PdgA, and was suspected to trigger exopolysaccharide synthesis via the Mxd machinery [24].

In this study, we first showed that the Bpf system is required for pellicle formation, but is dispensable for SSA-biofilm formation. We then proved that BpfD binds c-di-GMP via its degenerated EAL domain and is therefore an effector protein. Finally, we found that BpfD belongs to a complex regulatory network governing pellicle formation by interacting not only with CheY3 but also with two DGCs (PdgA and PdgB).

## 2. Results

### 2.1. The Bpf System Is Not Required for SSA-Biofilm Formation in S. oneidensis

**Figure 1:**
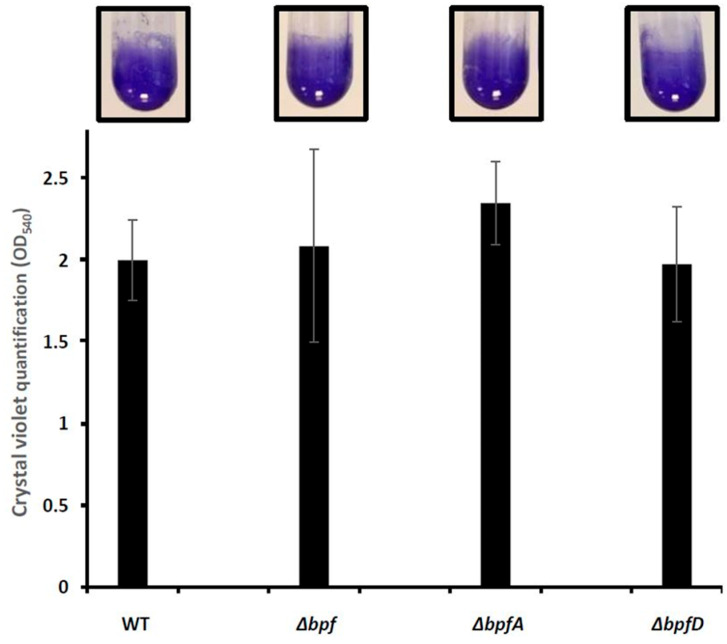
The *bpf* mutants of *Shewanella oneidensis* are able to form an SSA-biofilm. The wild-type MR1-R (WT), Δ*bpfA*, Δ*bpfD,* and Δ*bpf* strains were grown at 28 °C under agitation in LM. After 24 h of incubation, biofilm formation was evaluated by crystal violet staining, photographed, and quantified by OD_540_ measurements. The graphs represent the means and standard deviations from two independent experiments conducted in duplicate.

In order to test the involvement of the *bpf* gene cluster in biofilm formation, we constructed various deletion mutants starting from the *S. oneidensis* MR1-R strain (referred to as wild-type). The Δ*bpfA* and Δ*bpfD* strains are deleted of the *so_4317* and *so_4323* genes, respectively, while the Δ*bpf* strain is deleted of the entire cluster, i.e., from *so_4317* to *so_4323*. The three resulting strains were first tested for their ability to form an SSA-biofilm. The wild-type and mutant strains were grown in LM (Lactate Medium) under shaking conditions for 24 h. The cells adhered to the tube walls were stained with crystal violet (CV). As expected, the wild-type strain formed an SSA-biofilm in these conditions (Figure 1). Unexpectedly, the Δ*bpfA*, Δ*bpfD,* and Δ*bpf* mutants were also able to form an SSA-biofilm (Figure 1). CV quantification indicated that the biomass of the *bpf* mutants is similar to that of the wild-type. These results show that, in these conditions, the Bpf system is not required for SSA-biofilm formation in *S. oneidensis*.

### 2.2. The Bpf System Is Mandatory for Pellicle Formation in S. oneidensis

**Figure 2:**
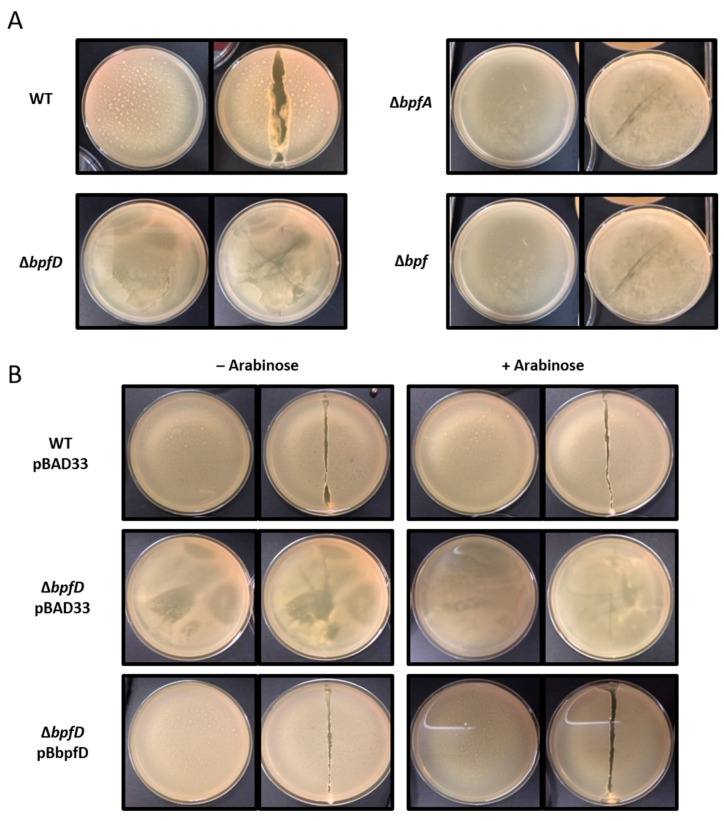
The Bpf system is involved in *Shewanella oneidensis* pellicle formation. (**A**) The wild-type (WT), Δ*bpfA*, Δ*bpfD,* and Δ*bpf* strains were grown at 28 °C in LB medium without agitation. (**B**) Strains containing either the pBAD33 vector or pBbpfD plasmid were grown at 28 °C in LB medium without agitation in the presence (+) or absence (−) of arabinose (0.2%). Pictures were taken after a 24 h incubation. For each strain, the pellicle phenotype was observed before (left panel) and after (right panel) disruption by a toothpick.

Since *S. oneidensis* is also able to form a pellicle, we wondered whether the Bpf system could be involved in this process. To test this, cells were grown in rich medium (LB) without shaking at 28 °C for 24 h. We then observed the formation of the pellicle and tested its integrity, thickness, robustness, and elasticity using a toothpick. As previously shown, the wild-type strain was able to form a mature and robust pellicle at the air–liquid interface (Figure 2A). On the contrary, the Δ*bpfA,* Δ*bpfD,* and Δ*bpf* mutants were unable to form a mature pellicle, suggesting that the Bpf system is mandatory for pellicle formation (Figure 2A). Strikingly, the Δ*bpfD* mutant showed a phenotype which was slightly different from that of the Δ*bpfA* and Δ*bpf* mutants. Indeed, pellicle fragments were observed on the edge of the plate, but the pellicle had not spread at the liquid surface (Figure 2A). It should be repeated that *bpfD* is the last gene of the *bpf* cluster, meaning that, in a Δ*bpfD* mutant, BpfA could be produced and exported and that only its cleavage is likely affected.

To ascertain that the defect of the Δ*bpfD* mutant is due to *bpfD* gene deletion and not to a polar effect, the *bpfD* gene was cloned under the control of an arabinose-inducible promoter in the pBAD33 vector, and the resulting pB-bpfD plasmid and the empty vector were then introduced into the Δ*bpfD* mutant. The strains were then cultured in the presence or absence of arabinose (Figure 2B). As expected, the presence of the pBAD33 vector did not modify the phenotypes of the wild-type and Δ*bpfD* strains. As shown in Figure 2B, the presence of the pB-bpfD plasmid restored pellicle formation in the Δ*bpfD* mutant, whether arabinose was added or not. This result confirms that the defect for pellicle formation observed in the Δ*bpfD* mutant was only due to the deletion of *bpfD*, meaning that BpfD is crucial for pellicle formation in *S. oneidensis*.

### 2.3. BpfD Acts as a c-di-GMP Effector

**Figure 3:**
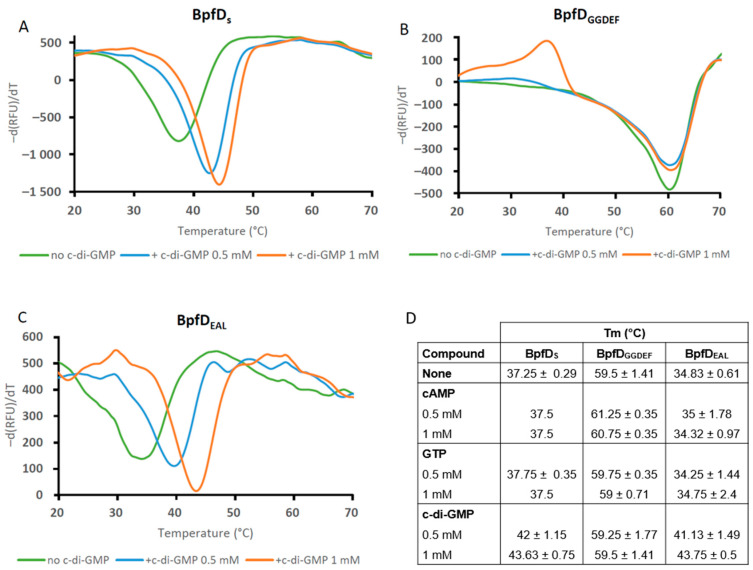
BpfD binds c-di-GMP via its degenerated EAL domain. Thermal shift assays (TSAs) were performed using the cytoplasmic part of BpfD (BpfD_S_) (**A**), its GGDEF domain (**B**), or EAL (**C**) domain and c-di-GMP, cAMP and GTP. The various domains of BpfD (7.5 µM) were incubated in the presence of SYPRO Orange and various concentrations of the different compounds. The mix was then submitted to a temperature gradient from 20 to 70 °C. Graphs represent the first derivative of the fluorescence emission (-d(RFU)/dT, RFU: Raw Fluorescence Unit) as a function of temperature. The melting temperatures (Tm) of each protein are listed in the table (mean values with standard deviation, *n* = 2 to 6) (**D**). All graphs are representative of two independent experiments.

BpfD is homologous to LapD. As LapD, it is predicted to be anchored to the membrane by two transmembrane segments and to possess a cytoplasmic part containing an HAMP domain, a degenerated GGDEF domain (AAFEF), and a degenerated EAL domain (ELY). We therefore wondered whether BpfD could bind the secondary messenger, cyclic di-GMP (c-di-GMP), and act as an effector protein of the Bpf system. To test this, we overproduced and purified the cytoplasmic region of BpfD containing the degenerated GGDEF and EAL domains (called BpfD_S_). We then performed thermal shift assays (TSAs) using purified BpfD_S_ alone or incubated with either c-di-GMP or other nucleotides (cAMP and GTP were used as control). The curve of the first derivative of fluorescence emission relative to temperature revealed one peak for BpfD_S_ alone with a melting temperature (Tm) of 37.25 °C (Figure 3A,D). When the TSA was performed in the presence of 0.5 mM and 1 mM c-di-GMP, the Tm of BpfD_S_ rose to 42 °C and 43.63 °C, respectively. The resulting ΔTm (Tm_protein + ligand_ − Tm_protein alone_) was therefore about 4.75 and 6.38 °C, respectively. When the TSA was carried out in the presence of cAMP or GTP, the Tm of BpfD_S_ remained unchanged compared to the condition without any ligand (Figure 3D). An additional control was performed using the CheY3 protein. The Tm of CheY3 was not changed by the addition of c-di-GMP (Figure S1). These results indicate that BpfD_S_ directly and specifically binds c-di-GMP.

To determine whether BpfD binds c-di-GMP via its degenerated GGDEF or EAL domain, we produced and purified the two domains independently (called BpfD_GGDEF_ and BpfD_EAL_, respectively). When BpfD_GGDEF_ was incubated alone, a Tm of about 59.5 °C was observed (Figure 3B,D). In the presence of either c-di-GMP or GTP, the Tm of this protein was unchanged (Figure 3D). A slight increase in the Tm was only observed in the presence of cAMP, but the ΔTm was below 2 °C, suggesting that there was no binding (Figure 3D). These results indicate that the degenerated GGDEF domain of BpfD is not able to bind c-di-GMP. However, when we performed similar experiments with BpfD_EAL_, an increase in the Tm was specifically observed in the presence of c-di-GMP. The ΔTm was about 6.3 °C and 8.92 °C in the presence of 0.5 mM and 1 mM c-di-GMP, respectively (Figure 3C,D). Altogether, these results show that BpfD binds c-di-GMP via its EAL domain.

**Figure 4:**
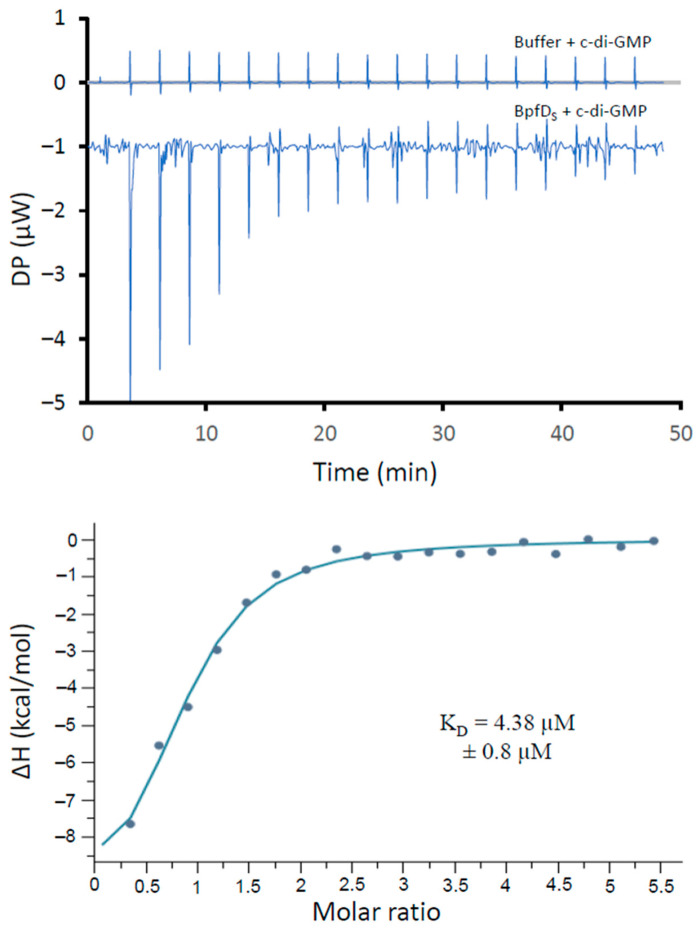
Interaction of BpfD_S_ with c-di-GMP tested by isothermal titration calorimetry. BpfD_S_ (20 µM) or dialysis buffer was submitted to several injections of c-di-GMP (575 µM). Top graphics show heat exchange upon ligand titration, either with dialysis buffer (control) or with BpfD_S_. The bottom graphic shows the integrated data after control subtraction with binding isotherms fitted according to a one-site binding model. The data shown are representative of two independent experiments.

To further validate the direct binding of c-di-GMP to BpfD and to estimate the affinity of this interaction, we performed isothermal titration calorimetry (ITC) with the purified BpfD_S_ protein. As shown in Figure 4, injection of c-di-GMP elicited an exothermic reaction, confirming that BpfD_S_ binds c-di-GMP. Data were fitted using the “One Set of Sites” model, and the apparent dissociation constant (*K_D_*) was calculated. BpfD_S_ binds c-di-GMP with a *K_D_* of 4.38 µM ± 0.8 µM, a value which is close to the estimated *K_D_* for the *P. fluorescens* LapD homolog (5.5 ± 2.8 µM) [26]. Altogether, TSA and ITC assays indicate that BpfD_S_ directly binds c-di-GMP and can act as a c-di-GMP effector via its degenerated EAL domain.

### 2.4. BpfD Interacts with the PdgA and PdgB Diguanylate Cyclases as Well as with the CheY3 Regulator

**Figure 5:**
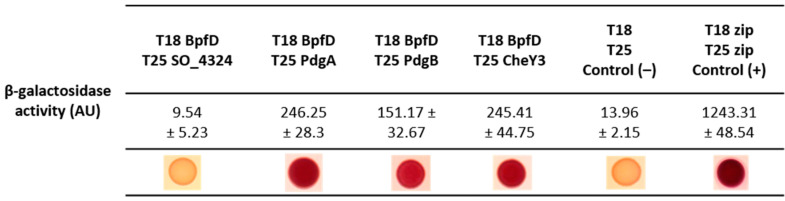
BpfD interacts with two diguanylate cyclases and the response regulator CheY3. *E. coli* BTH101 containing pUT18-BpfD and pKT25-SO4324, CheY3, PdgA, or PdgB were tested in this experiment. As controls, pEB354 (T25) with pEB355 (T18) (negative) and pT18-zip with pT25-zip (positive) were used. β-galactosidase activity was measured at 420 nm using a TECAN™ spectrophotometer after the addition of ONPG (4 mg.mL^−1^). Measures are indicated as mean values in arbitrary units (AUs) and their standard deviations. Values are representative of three independent experiments. The same strains were also spotted on MacConkey plates containing lactose and photographed after 48 h of incubation. All pictures were taken from the same plate and are representative of at least three experiments.

In the Lap system of *P. fluorescens*, the LapD protein was proved to physically interact with a specific diguanylate cyclase (GcbC) and proposed to receive c-di-GMP from GcbC [13]. We thus wondered whether this was also the case for BpfD. We therefore searched for a DGC partner of BpfD. First, we performed a Blast search in the genome of *S. oneidensis* using the sequence of GcbC as a query. This resulted in numerous diguanylate cyclases (44 hits), but did not pinpoint a close homolog with a similar domain organization. Second, we looked at the genetic context for the *gcbC* gene in *P. fluorescens* and found that it is close to the *ftsZ*, *ftsA,* and *ftsQ* genes. The homologs of these three genes in *S. oneidensis* are *so_4215*, *so_4216,* and *so_4217*, but no gene encoding a DGC was found close by. Interestingly, a gene encoding a putative diguanylate cyclase (*so_4324*) was found downstream of *bpfD* and separated from it by several tRNA genes. SO_4324 could be a good candidate as the DGC partner of BpfD. Moreover, as the Bpf system is mandatory for pellicle formation, we wondered whether the two DGCs (PdgA and PdgB) previously found to be involved in pellicle formation could be BpfD partners. We thus carried out bacterial two-hybrid assays using BpfD fused to the T18 domain of the adenylate cyclase, and the other proteins (PdgA, PdgB, SO_4324) fused to the T25 domain. After incubation on MacConkey–lactose plates, the cells producing T18-BpfD with either T25-PdgA or T25-PdgB turned red, while the cells producing T18-BpfD with T25-SO_4324 did not (Figure 5). Accordingly, β-galactosidase activities measured on the strains containing T18-BpfD and either T25-PdgA or T25-PdgB were significantly higher than those measured on the control strain containing the empty vectors or the strain containing T18-BpfD and T25-SO_4324 (Figure 5). These results suggest that PdgA and PdgB, but not SO_4324, interact with BpfD.

Since pellicle formation is controlled by a complex regulatory network centered around the chemotaxis regulator CheY3, we wondered whether BpfD could interact with CheY3. We thus performed a bacterial two-hybrid assay using T18-BpfD and T25-CheY3. As shown in Figure 5, the cells containing T18-BpfD and T25-CheY3 turned red on MacConkey–lactose plates and the β-galactosidase activity was significantly higher than that of the control strain, suggesting that BpfD interacts with CheY3.

**Figure 6:**
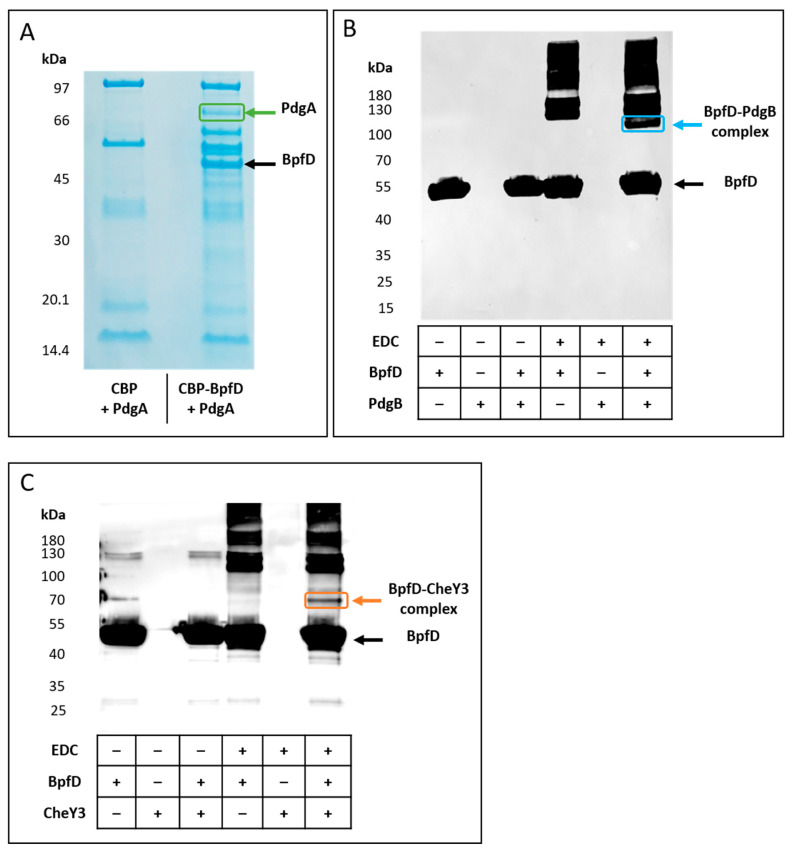
Interaction of BpfD with PdgA, PdgB, and CheY3. (**A**) Co-purification assay of BpfD and PdgA. PdgA was co-produced either with CBP-BpfD or CBP. CBP-BpfD (or CBP) was purified using CBP affinity resin, and bound proteins were submitted to SDS-PAGE. The band surrounded in green was excised and analyzed by mass spectrometry. The presence of PdgA was confirmed (coverage % = 60; PSM number = 315; unique peptides = 35). (**B**,**C**) Crosslinking experiments. Purified Strep-tagged BpfD_S_ and His-tagged PdgB (**B**) or Strep-tagged CheY3 (**C**) was incubated in the presence or in the absence of the crosslinker EDC (− indicates the absence of the proteins and EDC, while + indicates their presence). All samples were submitted to SDS-PAGE. After blotting, the membranes were revealed using anti-StrepTag II antibodies. Due to its low size, the Strep-tagged CheY3 is not detected in these electrophoretic conditions. The band surrounded in blue was analyzed by mass spectrometry. The presence of BpfD (coverage % = 73; PSM number = 220; unique peptides = 27) and PdgB (coverage % = 62; PSM number = 58; unique peptides = 17) was confirmed. The band surrounded in orange was analyzed by mass spectrometry. The presence of BpfD (coverage % = 66; PSM number = 125; unique peptides = 23) and CheY3 (coverage % = 65; PSM number = 46; unique peptides = 6) was confirmed.

To confirm these results, we first performed pull-down assays. To do so, the *bpfD* gene was cloned into the pBAD24-CBP-linker plasmid in which the *cbp* gene, encoding the calmodulin-binding protein, was placed under the control of the arabinose-inducible promoter. The resulting construction (pBcbp-bpfD) allows the production of a CBP-BpfD chimeric protein. An *E. coli* strain was then co-transformed with pBcbp-bpfD (or pBAD24-CBP-linker, used as control) and either pBpdgA, pBpdgB, or pBcheY3. The cells were then grown in the presence of arabinose, allowing the co-production of CBP-BpfD (or CBP only) with either PdgA, PdgB, or CheY3. Cell extracts were subsequently incubated with calmodulin-coated beads, allowing CBP-BpfD (or CBP) purification. The elution fractions were then submitted to SDS-PAGE and their protein contents were analyzed. When PdgA was co-produced with BpfD, a band was observed at a position which is in agreement with the molecular mass of PdgA (80 kDa), while this band was absent when the experiment was performed with CBP only (Figure 6A). The presence of PdgA was confirmed by a mass spectrometry experiment performed after excision of the band from gel (Figure S2 and Table S1). This result confirms that BpfD interacts with the diguanylate cyclase PdgA.

Unfortunately, pull-down assays performed using PdgB or CheY3 in combination with CBP-BpfD did not show co-purification of BpfD with either one of the two proteins. This could be due to a low level of detection or either transient or weak interaction. Therefore, in order to confirm the interaction of BpfD with PdgB and CheY3, we performed crosslinking experiments using Strep-tagged BpfD_S_, His-tagged PdgB, and Strep-tagged CheY3 purified proteins. As shown on Figure 6B,C, when BpfD_S,_ is incubated alone in the presence of the crosslinker, complexes of higher molecular masses are observed, suggesting that BpfD is able to multimerize. When BpfD_S_ and PdgB were incubated in the presence of the crosslinker (EDC), an additional band was observed below the multimeric forms of BpfD (Figure 6B). The molecular mass of this complex is between 100 kDa and 130 kDa and could correspond to a monomer of BpfD (50.5 kDa) interacting with a dimer of PdgB (2 × 35 kDa). The presence of both BpfD and PdgB in this complex was confirmed by mass spectrometry (Figure S3 and Table S1).

When BpfD_S_ and CheY3 were incubated in the presence of the crosslinker (EDC), an additional band was observed between the monomeric and the multimeric forms of BpfD (Figure 6C). The molecular mass of this complex is between 70 kDa and 100 kDa and could correspond to a monomer of BpfD (50.5 kDa) interacting with a dimer of CheY3 (2 × 16 kDa). The presence of both BpfD and CheY3 in this complex was confirmed by mass spectrometry (Figure S4 and Table S1).

Altogether, these results indicate that BpfD not only interacts with two diguanylate cyclases previously shown to be involved in pellicle formation but also with CheY3, which is at the center of a complex regulatory network controlling biofilm formation.

## 3. Discussion

**Figure 7:**
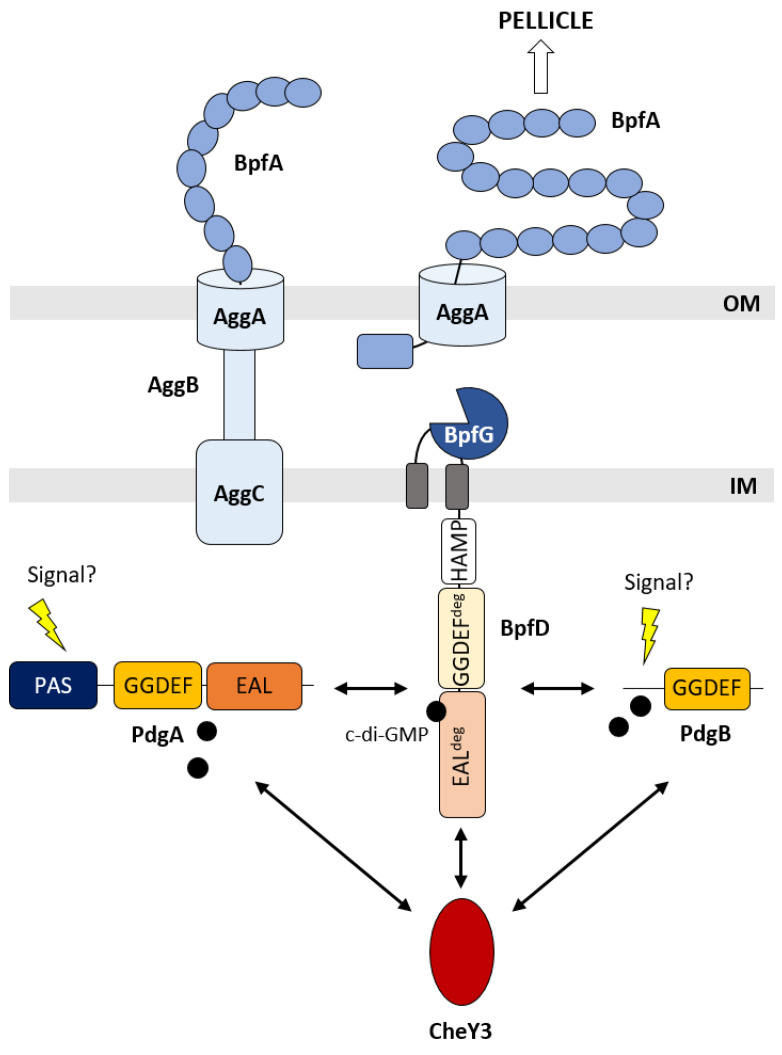
The Bpf system and its partners are involved in pellicle formation in *S. oneidensis*. BpfA is an adhesin secreted by a type-I secretion system composed of the AggA, AggB, and AggC proteins. BpfG is a periplasmic protease. BpfD is a membrane-anchored protein with an HAMP domain as well as degenerated GGDEF and EAL domains (GGDEF^deg^ and EAL^deg^). PdgA and PdgB are diguanylate cyclases and CheY3 is a response regulator proved to be mandatory for pellicle formation. Based on our results, we propose that BpfD is a c-di-GMP effector receiving the c-di-GMP secondary messenger from PdgA and PdgB, which could be activated by yet-unknown signals. BpfD also interacts with CheY3 and therefore belongs to the complex regulatory network controlling pellicle biogenesis. Fixation of c-di-GMP on the degenerated EAL domain of BpfD probably allows BpfD to sequester BpfG, leading to the accumulation of BpfA at the cell surface and pellicle formation. The protein–protein interactions, which were experimentally proven, are indicated by double arrows. IM: inner membrane; OM: outer membrane.

The Bpf system of *S. oneidensis* was proposed to be an ortholog of the Lap system of *P. fluorescens*. Indeed, in addition to sequence homologies and syntheny conservation, it was already shown that *bpf* mutants are either totally or partially impaired in biofilm formation and interactions between partners of the system are conserved [15,23]. However, several questions remained unanswered. (1) Is the Bpf system required for both pellicle and SSA-biofilm formation? (2) Is BpfD able to bind c-di-GMP, and if this is the case, is there a specific DGC delivering c-di-GMP directly to BpfD? (3) Is the Bpf system connected to the complex CheY3-centered regulatory network, which has been shown to control both pellicle and SSA-biofilm formation? Our study provides clues to answer these questions, as schematized in Figure 7.

First, we showed that the *bpf* mutants are still able to form SSA-biofilm but are either totally (Δ*bpfA* and Δ*bpf*) or partially (Δ*bpfD*) impaired in pellicle formation. Consistent with these results, a previous study reported that a mutant of *aggA* (*so_4320*), a BpfA type-I secretion system-encoding gene, is unable to form a pellicle [19]. This strongly suggests that the Bpf system is specific to pellicle formation. The difference in behavior between the Δ*bpfA* and Δ*bpfD* mutants is reminiscent of what was observed for the Δ*lapA* and Δ*lapD* mutants of *P. fluorescens*. Indeed, while the Δ*lapA* mutant is severely impaired for biofilm formation, the Δ*lapD* mutant is still able to form a biofilm somewhat different from the wild-type strain [27,28]. The authors proposed that the difference could be due to the fact that LapA protein is absolutely required for biofilm formation, while LapD only controls LapA secretion. This hypothesis could also apply to the Bpf system. Nevertheless, it should be mentioned that the role of BpfA is probably different from that of LapA. While LapA was shown to be involved in the interaction between the cells and the surfaces, BpfA does not seem to be necessary for adhesion to surfaces since the Δ*bpfA* mutant is still able to form an SSA-biofilm [29]. One hypothesis could be that BpfA is involved in cell–cell or cell–matrix interactions. Interestingly, BpfA and LapA, while both belonging to the RTX adhesion family, present different domain architectures, which could explain the difference in function. It is noteworthy that CdrA, an adhesin of *P. aeruginosa* different from LapA but also controlled by LapG, was shown to bind to the matrix Psl exopolysaccharide, leading to robust biofilms [30,31].

Second, we showed that the cytoplasmic domain of BpfD specifically binds c-di-GMP with an apparent *K_D_* value in the low micromolar range like its LapD counterpart. This interaction involves the degenerated EAL domain of BpfD, as also demonstrated for LapD [26]. BpfD is therefore acting as a c-di-GMP effector protein and probably controls the maintenance of BpfA at the cell surface by sequestering BpfG. An interaction between the periplasmic region of BpfD and the BpfG protein was indeed observed using bacterial two-hybrid experiments [15]. We then identified two DGCs physically interacting with BpfD, namely PdgA and PdgB, while no interaction was found with the SO_4324 DGC encoded by a gene close to the *bpf* operon. This makes sense, since PdgA and PdgB were identified to be involved in pellicle formation for which the Bpf system is mandatory [24].

Interaction between LapD and the DGC GcbC was shown to involve the α^2^ helix of the LapD EAL domain (α^2^-EAL:^462^GRFLPWLER^470^) and the α^5^ helix of the GcbC GGDEF domain (α^5^-GGDEF:^477^EQLLFAADK^485^) [13]. Interestingly, BpfD contains the sequence GQFMPYIEL at the position corresponding to the α^2^-EAL of LapD, while PdgA and PdgB contain GQLISLADT and EDTLKRADA, respectively, at the position corresponding to the α^5^-GGDEF of GcbC. It is therefore possible that these sequences are involved in the interaction between BpfD and its two partners PdgA and PdgB.

The fact that BpfD interacts with two DGCs is not so surprising. Indeed, a large-scale interaction study using bacterial two-hybrid experiments has shown that LapD of *P. fluorescens* could interact with 15 different partners, among which are 12 DGCs [32]. Each DGC could respond to a specific signal triggering its diguanylate cyclase activity. Actually, many DGC proteins encompass detecting modules such as PAS, Cache, GAF, CZB, etc. In the case of GcbC, a periplasmic Cache domain senses the presence of citrate [33]. Both PdgA and PdgB are predicted to be cytoplasmic proteins and have an *N*-terminal extension upstream of the GGDEF domain. While no known sensory domain is predicted in the N-terminal region of PdgB, a PAS domain is present in PdgA. Interestingly, CdgF from *Bacillus cereus* has a similar architecture to that of PdgA, i.e., PAS-GGDEF-EAL, and was shown to contain a flavin cofactor bound to the PAS domain. This bifunctional enzyme possesses a prominent diguanylate cyclase activity when the flavin cofactor is in the oxidized form, while the phosphodiesterase activity is upregulated when the PAS domain flavin cofactor is reduced [34]. We can imagine that PdgA could behave similarly and be active in oxygenated conditions, which is in good agreement with its role in biofilm formation at the air–liquid interface (pellicle).

Finally, we showed that BpfD also interacts with the CheY3 response regulator. The latter was previously demonstrated to be mandatory for both pellicle and SSA-biofilm formation, and proposed to be at the center of a complex regulatory network composed of several DGCs and a c-di-GMP effector protein (MxdA) [24,25]. It seems that this network is even more complex and includes another c-di-GMP effector protein, BpfD. This is reminiscent of the Hub-based model proposed for local c-di-GMP signaling [35]. Although interesting, many questions remain on how these Hub systems function, in particular whether or not they are modular depending on the environmental cues. In the case of *S. oneidensis*, we hypothesize that the network could be responsive to specific cues, since pellicle and SSA-biofilm-controlling networks seem to involve specific components but share a common knot, namely CheY3.

## 4. Materials and Methods

### 4.1. Strains and Growth Conditions

In this study, we used *S. oneidensis* and *E. coli* strains, which were routinely grown in lysogeny broth (LB) medium at 28 °C for *S. oneidensis* and at 37 °C for *E. coli*. Antibiotics were added when necessary: rifampicin (10 µg.mL^−1^), ampicillin (50 µg.mL^−1^), kanamycin (25 µg.mL^−1^), or chloramphenicol (25 µg.mL^−1^). All strains used in this work are listed in Table 1.

### 4.2. Construction of Deletion Mutants 

The Δ*bpfA*, Δ*bpfD*, and Δ*bpf* mutant strains were constructed as previously described [40]. Briefly, upstream and downstream regions flanking the gene (s) to be deleted were cloned into the suicide vector pKNG101. The ligation product was introduced into *E. coli* CC118 λpir. The resulting plasmid was introduced into the *S. oneidensis* MR1-R strain by conjugation using the *E. coli* helper strain 1047/pRK2013. The plasmid was integrated into the chromosome by a first recombination event and removed by a second recombination event in the presence of 6% sucrose. Deletions were confirmed by PCR (Figure S5).

### 4.3. Plasmid Constructions

All plasmids used in this work are listed in Table 2. To construct the plasmid pBbpfD, the entire coding sequence of *bpfD* (*so_4323*) was PCR-amplified using chromosomal *S. oneidensis* DNA as a template with primers containing the appropriate restriction sites and an optimized Shine Dalgarno. After digestion, the PCR product was inserted into the pBAD33 vector.

To construct the plasmids pETbpfD, pETbpfD_GGDEF_, and pETbpfD_EAL_, the coding sequence of *so_4323* (from position 225 to 639, from position 225 to 396, and from position 397 to 639, respectively) was PCR-amplified from *S. oneidensis* genomic DNA and cloned into pET52b with a sequence encoding a Strep-Tag upstream on the vector (Novagen).

For two-hybrid experiments, the *so_4324* coding sequence from *S. oneidensis* was cloned in-frame at the 3’ end of the sequence coding for the T25 domain of adenylate cyclase into pEB354, leading to pKT25-SO4324. The *bpfD* (*so_4323*) coding sequence (from position 225 to 639) was cloned in-frame at the 3’ end of the sequence coding for the T18 domain of adenylate cyclase into pEB355, leading to pUT18-bpfD.

To construct pBcbp-bpfD, the *bpfD* (*so_4323*) coding sequence (from position 225 to 639) was cloned in-frame at the 3’ end of the sequence coding for the calmodulin-binding domain into pBAD24-CBP-linker.

All constructs were checked by DNA sequencing using appropriate primers.

### 4.4. SSA-Biofilm Formation Assay

For SSA-biofilm formation, *S. oneidensis* cells were cultivated in poor medium under agitation as previously established [24,25]. *S. oneidensis* cells were first grown overnight on LB plates and resuspended in 10 mL of LB medium. They were then diluted in LM (Lactate Medium) (0.2 g.L^−1^ yeast extract, 0.1 g.L^−1^ peptone, 10 mM HEPES (pH 7.4), 10 mM NaHCO_3_ and 20 mM lactate) at an optical density (OD) of 0.05 at 600 nm. For each tested condition, 2 mL of cells was put into borosilicate glass tubes. Incubation was performed at 28 °C with shaking for 24 h. Each tube was then emptied, filled with 3.5 mL of 0.2% crystal violet, and colored for 10 min. The tubes were then rinsed several times with water, in order to remove unbound crystal violet, and photographed. Spectrophotometric quantification was then performed: crystal violet was solubilized in 3 mL of 30% acetic acid and OD_540_ was measured. Strains containing plasmids were grown overnight in the presence of chloramphenicol. 

### 4.5. Pellicle Formation Assay

For pellicle formation, *S. oneidensis* cells were cultivated in rich medium under static conditions as previously established [20,24]. *S. oneidensis* cells were first grown overnight on LB plates and resuspended in LB medium before being diluted in the same medium to reach an OD_600_ of 0.2. The suspensions were then transferred into Petri dishes and incubated for 24 h at 28 °C without agitation. Strains containing plasmids were grown overnight on plates containing antibiotics. When indicated, arabinose was added at 0.2%. Pictures were taken above the plates before and after the use of a toothpick on the pellicle surface.

### 4.6. Expression and Purification of Recombinant Proteins

Recombinant proteins Strep-BpfD_S_ (cytoplasmic part of BpfD only)_,_ Strep-CheY3, and PdgB-His were produced from *E. coli* BL21 (DE3) strains containing pETbpfD, pETcheY3, and pETpdgB, respectively. The strains were grown aerobically to reach an OD_600_ of 0.8. Overproduction of the proteins was then allowed by adding isopropyl-β-D-thiogalactopyranoside (1 mM) and incubating for 1 h (pETbpfD) or 3 h (pETpdgB and pETcheY3) at 37 °C. The cells were then collected by centrifugation (10 min at 8000 rpm and 4 °C), resuspended in 100 mM Tris-HCl pH 7.5 and 150 mM NaCl (Strep-BpfD/CheY3) or 20 mM phosphate buffer pH 7.4 (PdgB-His), disrupted by a French press, and centrifuged at 11,000 rpm and 4 °C for 15 min. The supernatant was then centrifuged at 45,000 rpm for 1 h at 4 °C. For the Strep-tagged proteins, the resulting supernatant was loaded on a Strep-Tactin resin (IBA), while a HisTrapFF resin (GE Healthcare) was used for PdgB-His. The recombinant proteins were purified according to the manufacturer’s protocol. Protein concentrations were estimated by Bradford assays (Bio-Rad).

### 4.7. Bacterial Two-Hybrid Assays

Bacterial two-hybrid experiments were performed as described by Battesti and Bouveret [37] with some modifications. Two-hybrid plasmids were co-transformed into the reporter strain *E. coli* BTH101 lacking the adenylate cyclase gene, and the clones were selected on LB agar containing 50 µg.mL^−1^ of kanamycin and 100 µg.mL^−1^ of ampicillin. For positive controls, we used pUT18-zip and pKT25-zip, while pEB354 and pEB355 were used as negative controls. The plates were incubated for 4 days at 28 °C. Ten isolated clones were then inoculated overnight in fresh LB with kanamycin, ampicillin, and 0.5 mM IPTG. Then, 2 µL of the cultures was spotted on MacConkey plates containing lactose (Difco™ MacConkey agar), kanamycin, and ampicillin. MacConkey plates were scanned after 48 h incubation at 28 °C. For β-galactosidase assays, cells were lysed by adding PopCulture Reagent solution (Agilent) and lysozyme at 1 mg.mL^−1^ for 15 min prior to adding Z buffer (100 mM phosphate buffer pH 7, 1 mM MgSo_4_, 10 mM KCl and 50 mM β-mercaptoethanol). Finally, 2.2 mM of ortho-nitrophenyl-β-galactoside (ONPG) was added. Afterwards, β-galactosidase activity was measured using a modified Miller assay adapted for use in a Tecan Spark microplate reader according to Baaziz et al. [44].

### 4.8. Thermal Shift Assays

For buffer exchange, purified proteins were loaded onto an NAP-5 desalting column (GE Healthcare) and recovered in 100 mM Tris-HCl (pH 7.5) containing 150 mM NaCl. Thermal shift assays (TSAs) were performed using a BioRad CFX96 Touch Real-Time PCR instrument. Samples were prepared in a total volume of 20 µL as described previously [24,45]. BpfD_S_ (7.5 µM), BpfD_GGDEF_ (7.5 µM), or BpfD_EAL_ (7.5 µM) were incubated in the presence of 10x SYPRO Orange (Sigma Life Science) with or without cAMP, GTP, or c-di-GMP (500 µM and 1 mM). Samples were then heated from 20 °C to 70 °C at a scan rate of 0.5 °C per 30 s. The protein unfolding curves were monitored by detecting changes in SYPRO Orange fluorescence. Melting temperatures were determined using the first derivative values of raw fluorescence data using Bio-Rad CFX Manager 3.1 software.

### 4.9. Isothermal Titration Calorimetry

The purified BpfD_S_ was dialyzed at 4 °C three times (for 1 h each) against Tris-HCl 100 mM (pH 7.4) and NaCl 150 mM. Isothermal titration calorimetry (ITC) experiments were performed using the MicroCal PEAQ-ITC (Malvern Panalytical, Palaiseau, France) at 25 °C. Then, 575 µM of c-di-GMP (Sigma-Aldrich, Saint-Quentin Fallavier, France) was titrated using 19 injections of 2 µL against 20 µM of BpfD_S_ in the sample cell of the ITC under a constant stirring speed of 750 rpm. C-di-GMP was also titrated against dialysis buffer and the resulting values were subtracted from the measured data with BpfD_S_. The PEAQ-ITC Analysis Software (version 1. 1. 0. 1262) was used to fit the collected data using a “One Set of Sites” model.

### 4.10. In Vivo Protein Interaction Assay 

Pull-down experiments were adapted from Battesti et al. [46]. *E. coli* C600 cells containing pBcbp-bpfD and pBpdgA were grown at 37 °C. At an OD_600_ of 0.8, 0.2% arabinose was added and the incubation was prolonged for 1 h. Cells were then centrifuged at 10,000 rpm for 10 min at 4 °C and resuspended in CBP buffer (10 mM Tris-HCl pH 8, 150 mM NaCl, 1 mM Mg acetate, 1 mM imidazole, 2 mM CaCl_2_, 0.1% Triton), disrupted by a French press, and pelleted at 8000 rpm for 10 min. The supernatant was incubated with 50 µL of calmodulin-binding peptide (CBP) affinity resin (Agilent) for 1 h on a stirring wheel. Then, the resin was centrifuged for 1 min at 2000 rpm, washed four times with 1 mL of the CBP buffer, resuspended in 40 µL loading buffer, and heat-denatured for 5 min at 95 °C. Proteins were then loaded on SDS-PAGE. A band excised from the gels was analyzed by mass spectrometry (LC-MS/MS).

### 4.11. In Vitro Protein Interaction Assay

Strep-tagged CheY3 (4.4 µM) or His-tagged PdgB (2.3 µM) were incubated with Strep-tagged BpfD_S_ (4.4 µM or 5.4 µM, respectively). Then, 5 mM of crosslinker 1-ethyl-3-(3-dimethylaminopropyl) carbodiimide hydrochloride (EDC) was added for 1 h 15 min at room temperature before stopping the interactions with Tris-HCl (1M pH 8). Interactions were then analyzed by Western blotting after SDS-PAGE using StrepTag II Antibody HRP Conjugate (Novagen). In parallel, the reactions were also loaded on SDS-PAGE and bands excised from the gel were analyzed by mass spectrometry (LC-MS/MS).

## Figures and Tables

**Table 1:** Strains used in the study.

Strains	Relevant Characteristics	Sources
*S. oneidensis* strains		
MR1-R	Rifampicin-resistant derivative of MR1	[36]
Δ*bpfA*	MR1-R deleted of *bpfA* (*so_4317*)	This work
Δ*bpfD*	MR1-R deleted of *bpfD* (*so_4323*)	This work
Δ*bpf*	MR1-R deleted from *bpfA* to *bpfD* (from *so_4317* to *so_4323*)	This work
*E. coli* strains		
BL21 (DE3)	F- *ompT hsdSB* (r_B_^−^m_B_^−^) *dcm gal* (DE3)	Novagen
BTH101	F- *cya-99 araD139 galE15 galK16 rpsL1* (Str^r^) *hsdR2 mcrA1 mcrB1*	[37]
C600	F- *tonA21 thi-1 thr-1 leuB6 lacY1 glnV44 rfbC1 fhuA1 λ−*	[38]
CC118 λpir	Δ*(ara-leu) araDE* Δ*lacX74 galE galK phoA20 thi-1 rpsE rpoB argE (Am) recA1 λpir*	[39]

**Table 2:** Plasmids used in the study.

Plasmids	Relevant Characteristics	Sources
pBAD33	Vector containing pBAD promoter with a p15A origin of replication (Cm^R^)	[41]
pBbpfD	Sequence coding for BpfD (SO_4323) cloned into pBAD33	This work
pBpdgA	Sequence coding for PdgA (SO_4552) cloned into pBAD33	[24]
pBpdgB	Sequence coding for PdgB (SO_0796) cloned into pBAD33	[24]
pBcheY3	Sequence coding for CheY3 (SO_3209) cloned into pBAD33	[20]
pET52b	Vector containing the T7 phage promoter and the coding sequence of StrepTagII (Ap^R^)	Novagen
pETbpfD	Sequence coding for the cytoplasmic part (S225 to E639) of BpfD (SO_4323) cloned into pET52b	This work
pETbpfD_GGDEF_	Sequence coding for the GGDEF domain (S225 to T396) of BpfD (SO_4323) cloned into pET52b	This work
pETbpfD_EAL_	Sequence coding for the EAL domain (E397 to E639) of BpfD (SO_4323) cloned into pET52b	This work
pETcheY3	Sequence coding for CheY3 (SO_3209) cloned into pET52b	[24]
pETpdgB	Sequence coding for PdgB (SO_0796) into pET21b	[24]
pEB355	pUT18C derivative, coding for the T18 domain of the adenylate cyclase of *Bordetella pertussis*	[37]
pUT18-bpfD	Sequence coding for the cytoplasmic part (S225 to E639) of BpfD (SO_4323) cloned in frame at the 3’ extremity of the sequence coding for the T18 domain into pEB355	This work
pUT18-zip	Sequence coding for a leucine zipper region cloned in-frame with the T18 domain (positive control)	[37]
pEB354	pKT25 derivative, coding for the T25 domain of the adenylate cyclase of *B. pertussis*	[37]
pKT25-SO4324	Sequence coding for SO_4324 cloned in frame at the 3’ end of the sequence coding for the T25 domain into pEB354	This work
pKT25-cheY3	Sequence coding for CheY3 (SO_3209) cloned in frame at the 3’ end of the sequence coding for the T25 domain into pEB354	[24]
pKT25-pdgA	Sequence coding for PdgA (SO_4552) cloned in frame at the 3’ end of the sequence coding for the T25 domain into pEB354	[24]
pKT25-pdgB	Sequence coding for PdgB (SO_0796) cloned in frame at the 3’ end of the sequence coding for the T25 domain into pEB354	[24]
pKT25-zip	Sequence coding for a leucine zipper region cloned in-frame with the T25 domain (positive control)	[37]
pBAD24	Vector containing pBAD promoter (Ap^R^)	[41]
pBAD24-CBP-linker	Sequence coding for the calmodulin-binding protein (CBP) cloned into pBAD24	[42]
pBcbp-bpfD	Sequence coding for the cytoplasmic part (S225 to E639) of BpfD (SO_4323) cloned in frame at the 3’ extremity of the sequence coding for the calmodulin-binding protein (CBP) into pBAD24-CBP-linker	This work
pKNG101	R6K-derived suicide plasmid containing Str^R^ and *sacB*	[39]
pRK2013	RK2-Tra1 RK2-Mob1 Km^R^ *ori* ColE1	[43]

## Data Availability

Data are contained within the article and Supplementary Materials.

## Supplementary Materials

The following supporting information can be downloaded at https://www.mdpi.com/article/10.3390/ijms25179697/s1.
