# Supplementary material for: BpfD Is a c-di-GMP Effector Protein Playing a Key Role for Pellicle Biosynthesis in Shewanella oneidensis
Source: Int J Mol Sci. 2024 Sep 7;25(17):9697. doi: 10.3390/ijms25179697 (PMC11395469; doi:10.3390/ijms25179697)
Supplement: Supplementary file 1 [file ijms-25-09697-s001.zip › Supplementary figures_ijms-3152329_Revision.pdf]

A

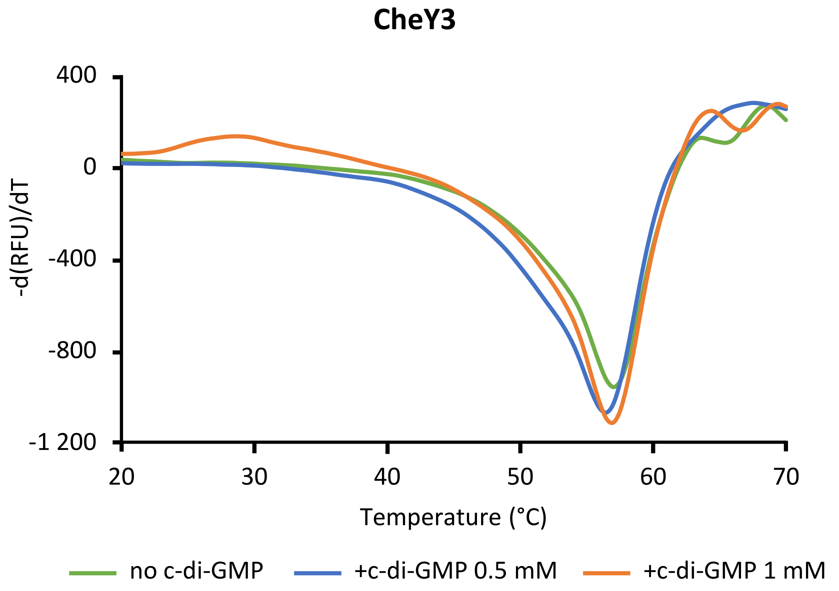

B

**Compound**	**Tm (°C)**
None	56.58 ± 1.96
c-di-GMP 0.5 mM	56.7 ± 1.15
c-di-GMP 1 mM	57.25 ± 0.35

Figure S1. CheY3 does not bind c-di-GMP. Thermal shift assays (TSAs) were performed using the Strep-tagged CheY3 protein (**A**). The CheY3 protein (7.5 µM) was incubated in the presence of SYPRO Orange in the absence or presence of c-di-GMP (0.5 and 1 mM). The mix was then submitted to a temperature gradient from 20 to 70 °C. Graphs represent the first derivative of the fluorescence emission (-d(RFU)/dT, RFU: Raw Fluorescence Unit) as a function of temperature. The melting temperatures (Tm) are listed in the table (mean values with standard deviation, n = 2 to 6) (**B**). All graphs are representative of two independent experiments.

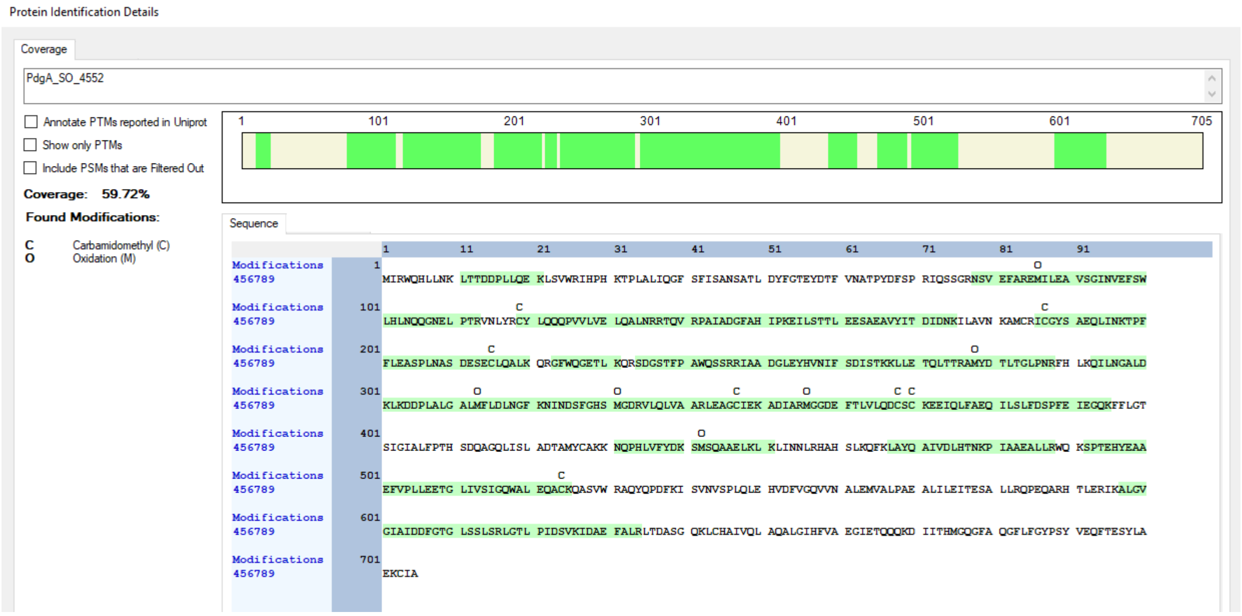

Figure S2. Identification of PdgA from gel-excised band (pull-down).
The band excised from the gel after the pull-down experiment (surrounded in green, Figure 6A) was digested by LysC and trypsin and then analyzed by LC-MS/MS.
The complete data are present in Table S1.

A

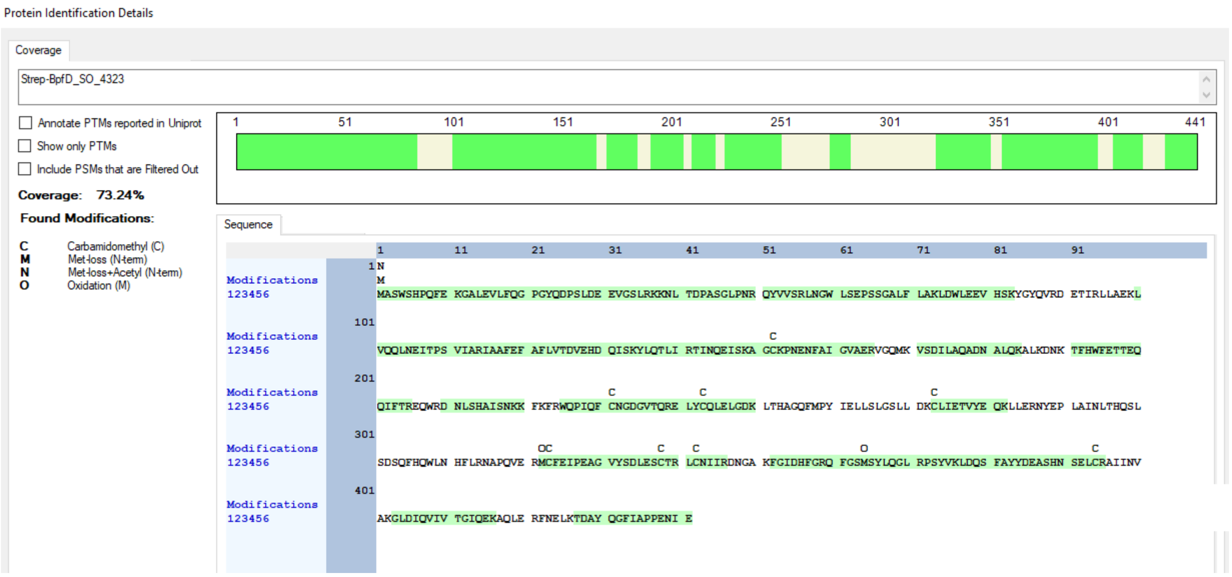

B

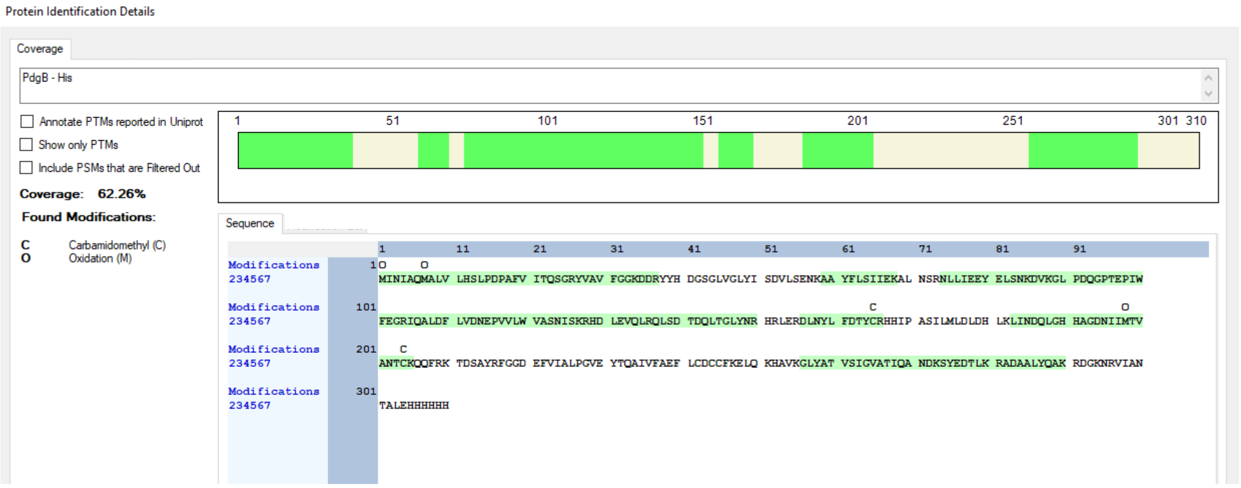

Figure S3. Identification of Strep-BfpD (A) and PdgB-His (B) from gel-excised band (crosslinking experiment).

The band excised from the gel after the crosslinking experiment (surrounded in blue, Figure 6B) was digested by LysC and trypsin and then analyzed by LC-MS/MS.

The complete data are present in Table S1.

A

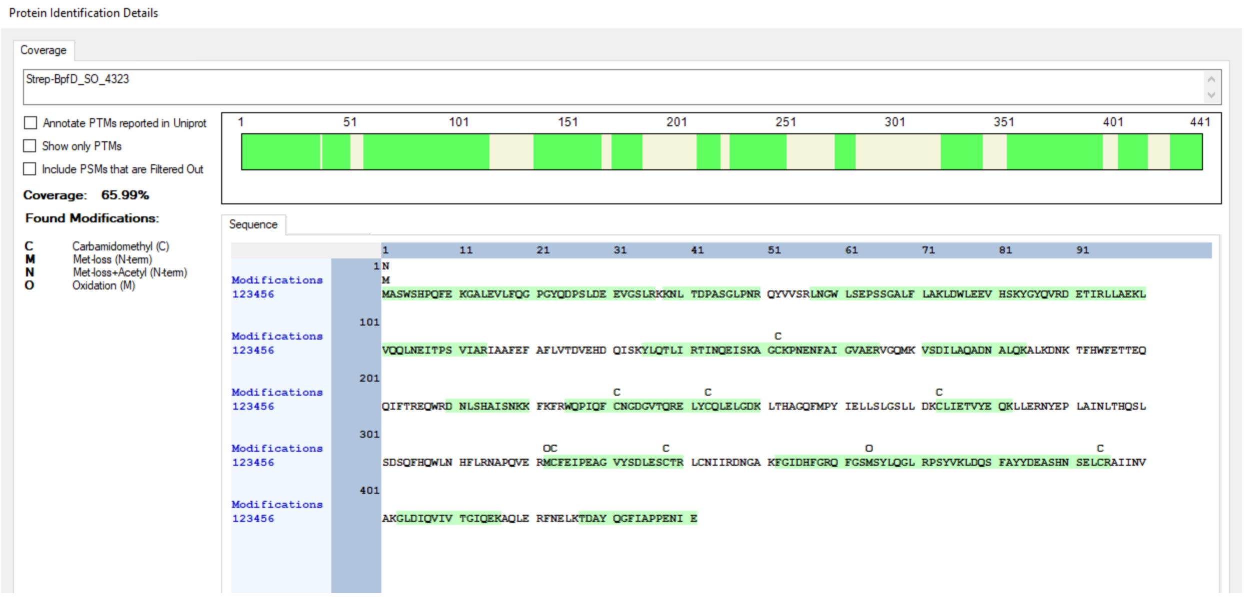

B

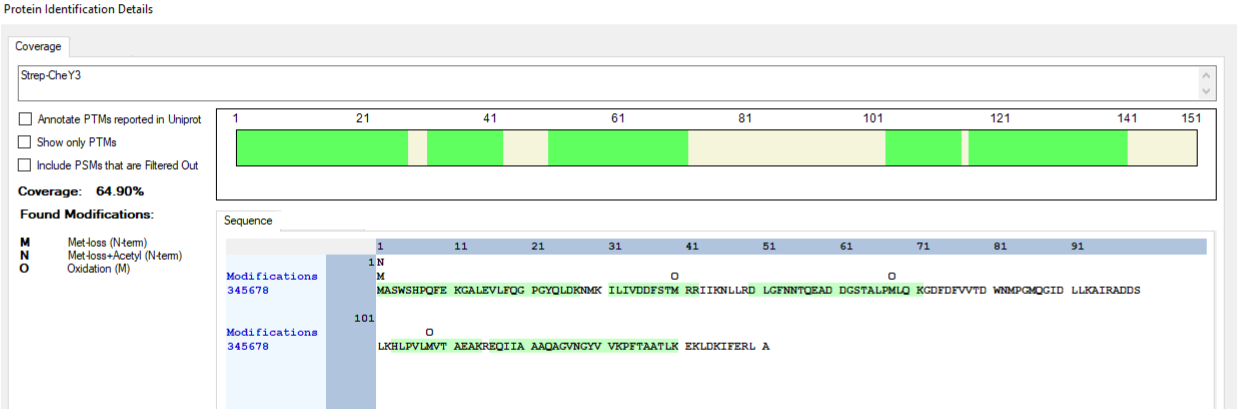

Figure S4. Identification of Strep-BfpD (A) and Strep-CheY3 (B) from gel-excised band (crosslinking experiment).

The band excised from the gel after the crosslinking experiment (surrounded in orange, Figure 6C) was digested by LysC and trypsin and then analyzed by LC-MS/MS.

The complete data are present in Table S1.

A

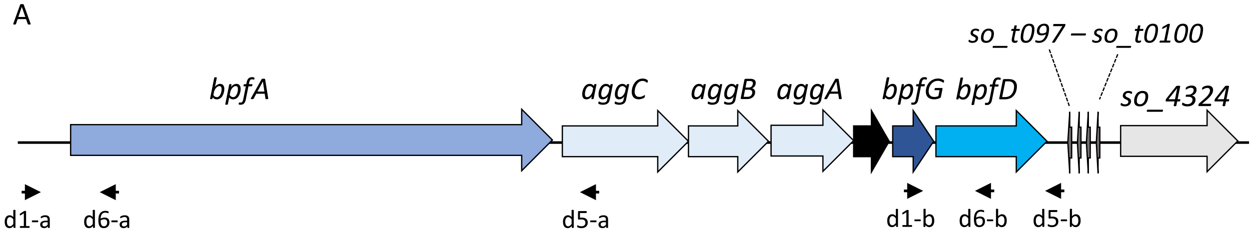

B

Primers / Strains	1 d1-a d5-a	2 d1-a d6-a	3 d1-b d5-b	4 d1-b d6-b	5 d1-a d5-b	6 d1-a d6-a
WT	9307 pb	932 pb	2998 pb	329 pb	17 636 pb	932 pb
ΔbpfA	**1134 pb**	**No amplicon**				
ΔbpfD			**1138 pb**	**No amplicon**		
Δbpf					**1134 pb**	**No amplicon**

C

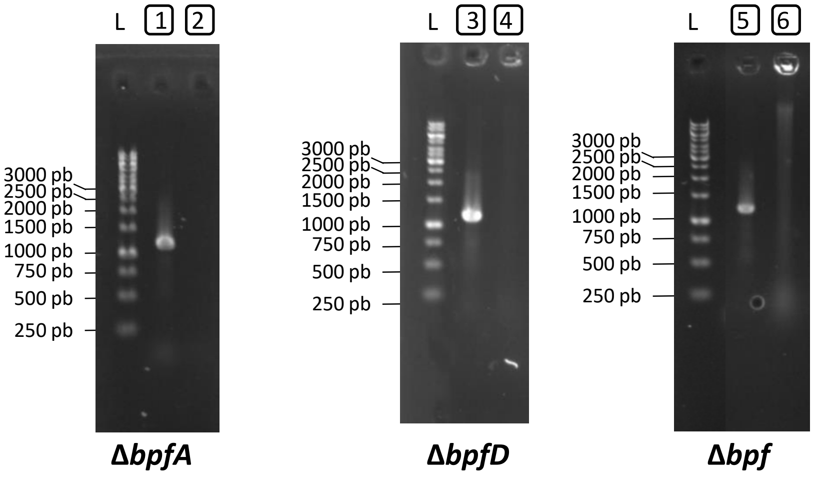

Figure S5. Confirmation by PCR of the *bpf* mutant strains.

The *bpf* locus is schematized. The different primers used for PCR are represented by black arrows below the genes (A). The sizes of the expected amplicons (when amplification is possible) are indicated in the table for the different strains (wild-type and mutants) and for the different couples of primers (B). The amplicons were visualized after electrophoresis on an agarose gel (C).
